# Ultra-Wideband-Based Time Occupancy Analysis for Safety Studies

**DOI:** 10.3390/s23177551

**Published:** 2023-08-31

**Authors:** Salah Fakhoury, Karim Ismail

**Affiliations:** Department of Civil and Environmental Engineering, Carleton University, 1125 Colonel By Dr., Ottawa, ON K1S 5B6, Canada; salah.fakhoury@carleton.ca

**Keywords:** wireless sensors, ultra-wideband (UWB), traffic conflicts, pedestrian safety

## Abstract

This study investigates the use of ultra-wideband (UWB) tags in traffic conflict techniques (TCT) for the estimation of time occupancy in a real-world setting. This study describes UWB technology and its application in the TCT framework. Many experiments were conducted to evaluate the accuracy of the occupancy time measurement using a UWB-based tag. The UWB performance was measured using data from UWB tags as well as a video camera system by subtracting the time occupancy within a conflict zone. The results show that the UWB-based system can be utilized to estimate occupancy time with a mean absolute error difference from ground truth measurements of 0.43 s in the case of using two tags and 0.06 s in the case of using one tag in an 8 m × 8 m study area with double-sided two-way communication. This study also highlights the advantages and limitations of using UWB technology in TCT and discusses potential applications and future research directions. The findings of this study suggest that the UWB-based localization of multiple tags needs further improvements to enable consistent multi-tag tracking. In future work, this technology could be utilized to estimate post-encroachment time (PET) in various traffic scenarios, which could improve road safety and reduce the risk of collisions.

## 1. Introduction

### 1.1. Background

Ultra-wideband (UWB) technology is increasingly being adopted for applications that involve localization and tracking, such as asset tracking and indoor positioning. UWB technology uses short-range radio waves to measure the time it takes a signal to travel between two devices. The accurate location data of road users is also crucial in developing modern technologies of far-reaching functionality such as the Internet of Things. A distinctive feature of a UWB signal is that it possesses various frequency components due to its broad bandwidth. Moreover, UWB does not usually disrupt other radio systems because of its low power spectral density. UWB offers high accuracy ranging and performs well under multipath conditions due to its short pulse duration [1]. Because of the accurate positioning data which can be obtained using UWB, it is promising to utilize in applications of traffic safety and, in particular, pedestrian safety. There is a persistent need to ensure the safety of drivers, as well as vulnerable road users in all weather conditions as well as during poor illumination. Therefore, UWB tags and anchors have the capacity to provide the real-time, accurate positioning of pedestrians, vehicles, and other objects in the road environment. Their reliance on high-frequency wireless signals could enable positioning when visibility is limited due to adverse weather conditions and light conditions.

Many applications, such as asset tracking and indoor positioning, have utilized UWB technology because of its reliability, scalability, and accuracy. In traffic applications, UWB tags could potentially be attached to vehicles and pedestrians. For positioning applications, UWB technology requires the installation of three or more stationary anchors that continuously measure the distance to a mobile UWB tag. These anchors can be placed along the roadside on common fixtures such as traffic light posts. Laadung et al. [2] noted that UWB distance measurements were calculated using Time of Flight (ToF), which estimates the time that a signal takes to travel from initiator to receiver. Moreover, typically ToF measurements can be achieved using single-sided two-way ranging (SS-TWR), double-sided two-way ranging (DS-TWR), or using time difference of arrival (TDoA).

**Figure 1:**
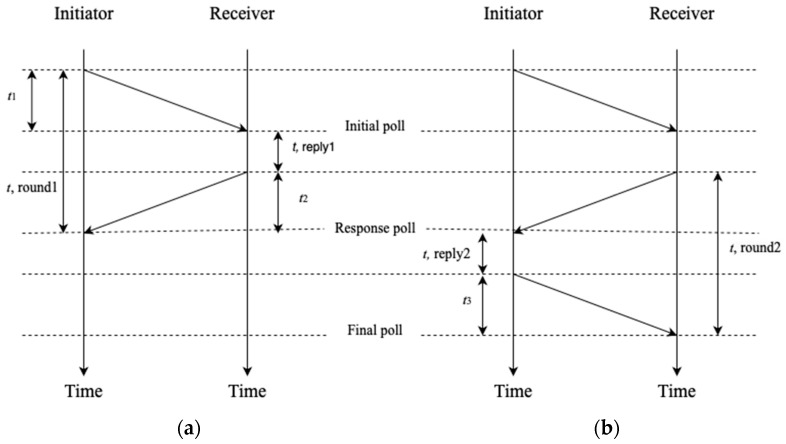
(**a**) Single-sided two-way ranging (SS-TWR). (**b**) Double-sided two-way ranging (DS-TWR).

The ranging techniques TDoA, SS-TWR, and DS-TWR are commonly used in many applications such as tracking systems and wireless communication to estimate distances or positions among UWB devices (e.g., anchors and tags). TDoA is a technique for ranging or positioning by measuring the time difference at which a signal arrives at several receivers. Furthermore, by comparing the time differences and applying the known positions or receivers, the distance or position estimation can be estimated. Figure 1 shows the difference between SS-TWR and DS-TWR. The ToF can be estimated in the SS-TWR by initially transmitting a signal from initiator to receiver. The receiver records its time of transmission *t*1. Subsequently, the receiver responds back to the initiator and records the time it took for a response to arrive *t*2. Finally, the ToF can be calculated as shown in Equation (1) [3,4,5].

(1) $$ToF\;\left(SS-TWR\right)=\frac{t,\;round1-t,reply1}{2}$$

Double-sided two-way ranging (DS-TWR) is a more accurate ranging method, compared to SS-TWR, as UWB devices transmit signals to each other. To estimate the distance between the initiator and responder, the initiator starts *t*, *round*1 by transmitting a signal to the receiver. Then, the receiver replies with a transmitted signal containing information about *t*, *reply*1, and starts *t*, *round*2. The response signal is received by the initiator, ending *t*, *round*1. The initiator replies with a final transmitting signal containing the *t*, *reply*2, which is the time the initiator takes to send the final signal back to the receiver. Finally, once the receiver receives the final signal, *t*, *round*2 ends, and the distance can be estimated [3,4,5].

(2) $$ToF\;\left(DS-TWR\right)=\frac{\left(\left(t,\;round1-t,reply1\right)+\left(t,\;round2-t,reply2\right)\right)}{4}$$

### 1.2. Literature Review

#### 1.2.1. Location-Based Applications of Wireless Sensors

Much research has investigated utilizing wireless sensors, including Bluetooth Low Energy, Bluetooth, and Wi-Fi, specifically when utilizing the information pertinent to localizing the emitting beacon. A previous study by Filippoupolitis et al. [6] looked at the use of Bluetooth Low Energy (BLE) for occupancy detection in the emergency management field. For instance, this study used BLE beacons to analyze and track the movements of an individual within a given area. As a result, the technology showed real-time occupancy information, which could potentially enhance emergency preparedness. Another study by Trivedi et al. [7] demonstrated the use of multiple wireless sensors, including BLE, Wi-Fi, and fused these to develop a system that could determine occupancy. The research showed 87.69% accuracy in occupancy detection.

Previous studies have investigated the use of Bluetooth Classic, Wi-Fi and BLE wireless sensors in collecting traffic studies for the localization data of road users. The research explored the utilization of the received signal strength indicator (RSSI) from the wireless sensors transmitted by beacons, or devices equipped with wireless sensors. First, the authors evaluated the performance of wireless sensors for vehicle–pedestrian collision warning systems. The study investigated the factors that could potentially affect the wireless sensors, such as motion effects, non-line of sight issues, including rainfall effects, the RSSI–distance relationship, and signal transmission rates. Based on the experimental results, the study found that the BLE mode illustrated better performance compared to Bluetooth Classic and Wi-Fi. Finally, the authors developed a technique for classifying and detecting turning movements in intersections using BLE signals transmitted by vehicles. The study analyzed the time profile of RSSI and compared signature points. The method showed 94.2% accuracy [8,9].

#### 1.2.2. Studies Using Ultra-Wideband (UWB)

Ultra-wideband has been noted as useful in high-accuracy localization applications. For example, it is useful for real-time safety monitoring to estimate the poses of heavy construction equipment at road construction sites. Researchers in previous works have developed systems (ViPER and ViPER+) to look at the use of UWB technology in pose estimation. Both studies investigated UWB performance by evaluating UWB technology with line-of-sight (LOS) and non-line-of-sight (NLOS) scenarios in a construction sites environment. The research introduced an input correction technique to reduce the impact of NLOS signals. For instance, the system applies a low-pass filter, reference, and anchor selection method, which chooses one anchor to broadcast the reference time. The ViPER pose estimation system resulted in a 117% increase in the packet reception ratio and a 70% reduction in the error rate compared to existing solutions in real-world parking lots and construction sites. ViPER+ used multiple tags to accurately determine the boundary of heavy construction equipment. The outcomes of ViPER+ demonstrated enhancement with a 40% improvement in localization accuracy and a 25% increase in the update rate compared to the ViPER system [10,11].

Dardari et al. [12] used UWB technology to study cyclist safety. UWB tags and anchors were used to track the movements of vehicles and bicycles at an intersection. Furthermore, Dardari et al. [12] claimed that the localization error was less than 50 cm in most static and dynamic condition cases. Therefore, time occupancy experiments were conducted to evaluate the distance and position accuracy in stationary and dynamic scenarios. Zhang et al. [13] explored the possibility of mounting UWB anchors on vehicles to localize nearby UWB tags. The proposed technique allows vehicles to locate a tag without relying on the fixed installation of anchors on roadside infrastructure. The experiments resulted in a 1.0 m error in estimating the horizontal distance between the moving anchors and nearby tags. This distance error was relatively high, which could form an obstacle for applications with higher accuracy demand.

Fakhoury and Ismail [14] evaluated the use of UWB in pedestrian safety. The study aimed to enhance pedestrian safety by estimating time-to-collision (TTC). Moreover, the study explored the potential use of UWB in estimating vehicle speed in real-world situations. The UWB beacon has a detection range of up to 25 m with a 3 cm error accuracy. TTC was analyzed in different speed vehicle scenarios to identify conflicts at various severity levels. The research showed the accuracy of measurements of UWB technology in various weather conditions. The findings of this research showed how time-to-collision estimation could reduce the risk of an accident by alerting pedestrians and vehicles by sending a notification to a smartphone and a flash from the phone to alert drivers.

#### 1.2.3. Traffic Conflict Techniques

The traffic conflict techniques (TCT) are used to assess traffic safety based on the observation of near-misses or near-collision events. Post-encroachment time (PET) has been used to estimate the level of severity of a traffic conflict. PET refers to the time available between two road users occupying a particular area, such that the time elapses between the moment the first road user exits the area, and the second road user arrives in the same area [15]. Many studies looked into pedestrian–vehicle interactions to estimate PET. For example, Whitley [16] investigated the use of LiDAR sensor trajectory data to estimate TTC and PET. However, the profile of this object could be affected by weather conditions, such as snow and rain [17]. Research by Kathuria et al. [18] explored the use of video cameras to analyze road user interactions between the pedestrian–vehicle and estimate PET. Nevertheless, traffic data collection could be affected by the position of the camera and resolution [19].

### 1.3. Study Objective

The objective of this study is to investigate the use of UWB in traffic safety techniques. In previous work, Fakhoury and Ismail [14] proposed techniques for TTC estimation. Moreover, pedestrian safety could be enhanced by exploring other traffic conflict indicators which does not require collision course, such as PET. This research conducted many preliminary experiments intended to estimate PET. However, the results revealed that the range accuracy can be affected by the range and signal interference from other second tags and cause delays for the first tag in transmitting signals. Gupta and Mohapatra, and August et al. [20,21] mention that the collision in transmission refers to a situation where multiple devices that are UWB-equipped transmit their signals simultaneously and interfere with each other, which results in data corruption. Limitations were faced in multi-tag positioning accuracy and reliability. Therefore, the experiments analyze time occupancy by using one tag and two tags with a collision in transmission.

## 2. Materials and Methods

### 2.1. Hardware Configuration

UWB tags are wireless devices whose locations can be determined through communication with fixed sensors or anchors using ultra-wideband technology. UWB tags offer a relatively high accuracy distance measurement because they can transmit and receive signals to allow more precise measurements of the time of flight and, hence, the distance. Furthermore, UWB tags are light and relatively inexpensive, thus offering flexible deployment because they can readily attach to moving objects such as vehicles, bicycles, or pedestrians. For example, in this study, a single UWB tag cost approximately USD 175 in 2022 and weighed approximately 35 gm. The cost paid for the kit was approximately USD 2000 in 2022, including four anchors and two tags. UWB tags are more suitable for applications that estimate object positions in motion because of their relatively high detection rate. In contrast, UWB anchors are heavier, weighing approximately 120 gm. Anchors are typically kept at fixed locations during data collection. The UWB anchors and tags work together to provide high-accuracy ranging data, with the UWB tags periodically exchanging signals with the anchors. Typically, a signal is emitted first by the anchor and subsequently travels in the atmosphere until it becomes detected, received, and sent back by the tag. The UWB sensors used in this study were developed by Estimote, Inc. in Kraków, Poland. The software implementation and coding presented in this study were developed in the Estimote-integrated development environment.

### 2.2. Distance Measurement

In general, the anchors can be placed at fixed locations, preferably in a grid pattern, by affixing them to a tripod or attaching them to a stable structure such as a wall, a ceiling, or a streetlight post. The UWB tags can be attached to a moving host, e.g., a vehicle or a pedestrian. Once the anchors and tags are secured in place, the UWB anchor exchanges signals with the tags. The range of the tag, measured relative to the anchor position, is calculated in terms of the signal’s time of flight. In this study, each anchor was affixed to a stable mounting device at a height of 0.91 m. Each anchor periodically communicated with the nearby tag to range the relative distance between them. This created a time sequence of distance measurements for each anchor-tag pair. This represented the data component registered at each anchor. In the early stages of the experiments, the distance data acquired by the tags were uploaded to cloud-based storage every 200 milliseconds. Specifically, this entailed selecting the most recent data gathered by the tag for each anchor and transmitting it to the cloud. This method was adopted because uploading data too often could result in a loss of data packets. In subsequent stages of the experiments, the data transmission was refined to aggregate detections into batches of 60 before uploading to the cloud. This modification allowed for a longer time between instances of uploading while maximizing the number of captured detections. Those data components were continuously updated as long as the UWB was enabled on both the anchor and the tag and provided that they were close enough to each other to successfully exchange the UWB signal. Once the UWB devices were disabled, the data analysis phase started. The stored data were downloaded as a structured text file in the format of JavaScript Object Notation (JSON).

The data consisted of distances and corresponding timestamps with the identification of the ranging anchor using the device’s fingerprint. Some filters were applied to the data in order to avoid noise. For example, although the experiments did not involve high-speed objects, some detections indicated high relative speeds due to inaccuracy in distance measurements and/or timestamp registration. To determine the speed of a tag relative to an anchor, the difference between two distance measurements, i.e., the displacement relative to the anchor position, was divided by the difference between their corresponding timestamps, i.e., the time interval between the two detections. If the measured speed exceeded a predefined threshold of 5 m/s, the entire detection, i.e., both the measured distance and the corresponding timestamp, meant that the measurement dropped. The timestamp was determined by the tag’s clock. Every measurement transmitted by the tags contained a UNIX timestamp indicating when the distance measurement was taken relative to an anchor, as determined by the tag’s clock.

After initial trials, it was found that the precise moment of distance measurement for an anchor-tag pair does not always coincide with all other anchors present in the study communicating with the same tag. This renders multilateration almost impossible because it requires simultaneous distance measurements. A method was developed to address this challenge by using interpolation over time to obtain all intermediate positions. This method was implemented in the Python language, which read the previously defined JSON file as the input. The purpose of this customized solution was to ensure that if a measurement for a particular anchor was not synchronous to one or more of the other anchors, then interpolated positions could be calculated for these asynchronous anchors.

First, the data component for each anchor was sequentially checked for any missing distance measurements at each timestamp, irrespective of which anchor was measuring this distance. That is, the method proceeded incrementally from one timestamp to another as long as each of the time stamps was reported by at least one anchor. For each asynchronous anchor(s), which had no distance measurement at the current timestamp, interpolation was performed. For each one of those asynchronous anchors, the previous and the next detections were selected as the endpoints of interpolation. The relative speeds of the tag, with respect to an anchor, at each of the two endpoints were calculated using a change in distance and the corresponding time interval at each endpoint. Then, the average of these two speeds was obtained, and it was assumed to be the constant speed of movement of the tag between the two endpoints. Subsequently, the missing distance measurement was estimated at the current moment by multiplying this average speed by the time that elapsed since the tag`s last known measurement, i.e., the earlier endpoint. If there were no measurements taken by the anchor before or after the current moment, the interpolated distance was not calculated and recorded as “null”. Finally, the tag`s absolute position, i.e., *x* and *y* coordinates in a local coordinate system, was calculated for each data point through multilateration, provided that there were enough simultaneous distance measurements available to perform this. However, if there were not enough measurements, the coordinates were not calculated, and each was recorded as “nan”.

**Figure 2:**
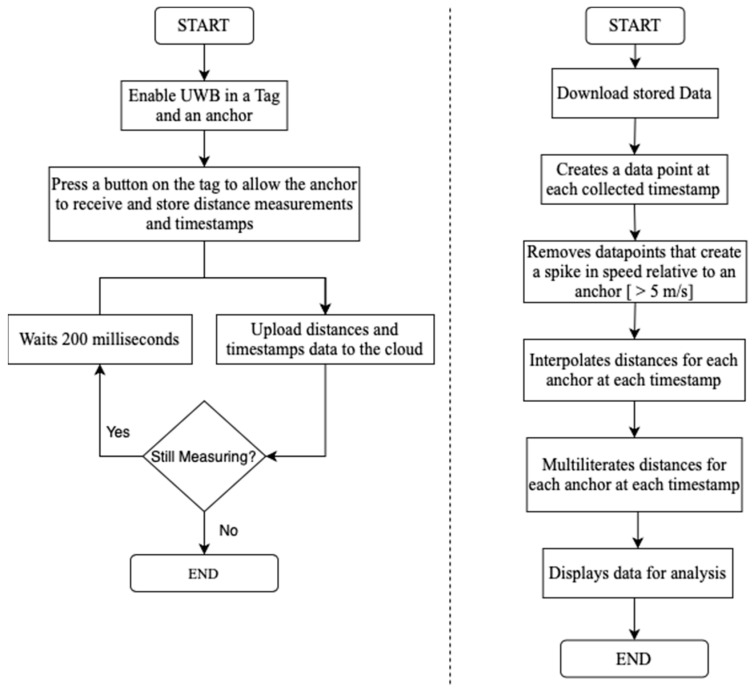
The left flowchart describes the data collection and recording processes. The right figure shows the developed method to impute for missing distance measurements.

The data recording process, as well as the previously described Interpolation method, are depicted in the following Figure 2.

## 3. Experiments

### 3.1. Static Positioning Experiments

**Figure 3:**
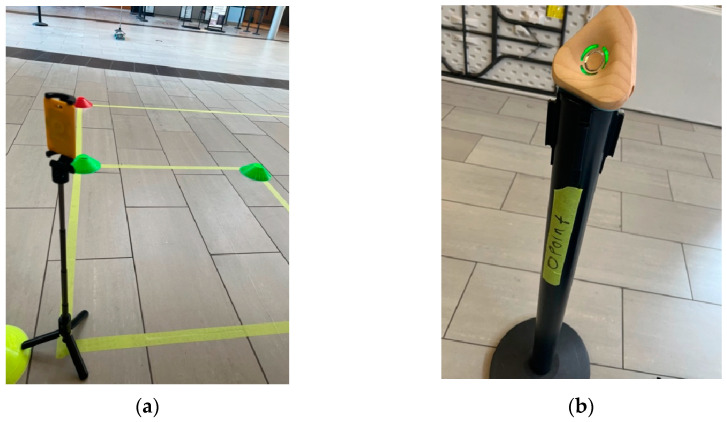
(**a**) A tag placed at one of the reference points; (**b**) A UWB anchor position.

This study aimed to examine the performance of UWB sensors for the purpose of detecting the time proximity between two tags as they cross each other’s path. As a preliminary examination, an experiment was conducted to evaluate the accuracy of the position calculation when the UWB tags were stationary. The experimental setup and used equipment are shown in Figure 3. Four anchors were used in the experiment to estimate the positions of the UWB tag. The anchors were placed at the corners of a 10 m by 10 m square study area at a fixed distance from each other.

Two stationary-tag experiments were conducted on the 7th and 15th of February, 2023. On 7 February 2023, a conflict zone measuring 2 m by 2 m was outlined on the floor, as illustrated in Figure 3a, diagonally from points with local coordinates (1,3) to (3,5). Another square conflict zone was outlined as 1 m by 1 m. A tag was set up for one minute at each of the following six reference points: (1,3), (2,3), (3,3), (1,4), (2,4), (1,5). The tag was also set up at the reference point (3,5), but these data were not uploaded properly to the server due to a technical issue. Table 1 shows the results of the average error in the location error at each reference point. The UWB tags were attached to a selfie stick at 0.65m height and were placed at each reference point, as demonstrated in Figure 3a. Furthermore, Figure 3b illustrates the anchors’ set up at 0.91 m height.

**Figure 4:**
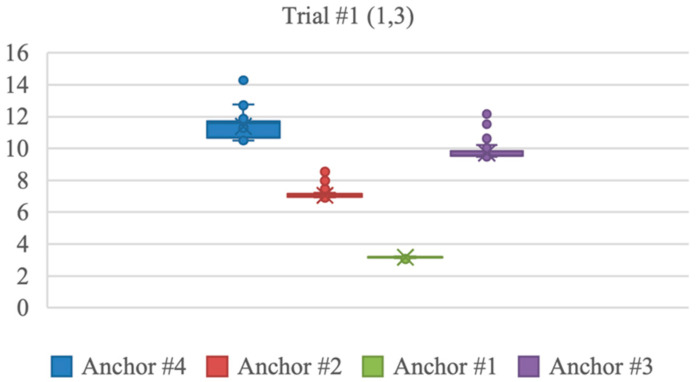
Distance measurements from each anchor at trial 1.

**Figure 5:**
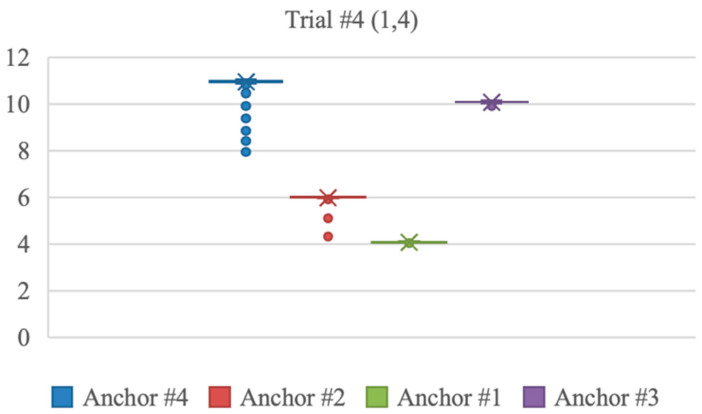
Distance measurements from each anchor at trial 4.

The *x* coordinate measurements had an average error of 0.03 m, excluding trials 1 and 4, where the error was notably higher, measuring 0.25 m and 0.22 m, respectively. The *y* coordinate measurements had an average error of 0.04 m across all trials. The distribution of the distances measured from the stationary tag to each anchor in trials 1 and 4 are shown in the following Figure 4 and Figure 5. Anchors #1 to 4 were located in the following order, anchor 1 at (0,0), 2 at (0,10), 3 at (10,0), and 4 at (10,10). The figures indicate that anchor 4 at (10,10) detected variable distance measurements, which caused positioning errors. In addition, anchors 2 and 3 at (10,0) and (0,10) also showed some measurement errors.

**Figure 6:**
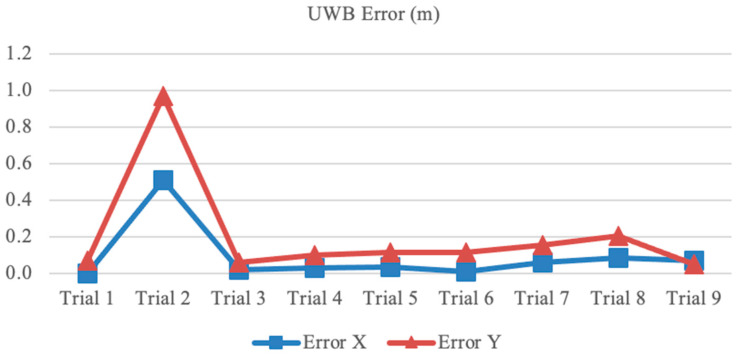
Positioning mean error.

On 15 February 2023, a stationary-tag experiment was conducted using nine reference points located at (1,1), (2,1), (3,1), (1,2), (2,2), (3,2), (1,3), (2,3), and (3,3) to analyze positioning accuracy. The UWB tag was placed at each reference point for 1 min, with each trial conducted at a different reference point. Sample raw data from one of the experiments are presented in Table 2, and the results from all nine trials are shown in Figure 6.

The average distance error measurements were 4 cm and 11 cm for the *x* and *y* coordinates, respectively, excluding the second trial at the (2,1) location. In the second trial, the distance error measurement was 0.97 m for the *y* coordinate and 0.51 m for the *x* coordinate. Several factors could have contributed to the distance error observed in the second trial, including the orientation of the anchor’s antennas, as suggested by Grasso et al. [22]. Their study found that positioning accuracy could be improved by directing the anchor’s antennas toward the center of the area being covered, resulting in distance errors of 5 cm for dynamic measurements and 15 cm for static measurements.

**Figure 7:**
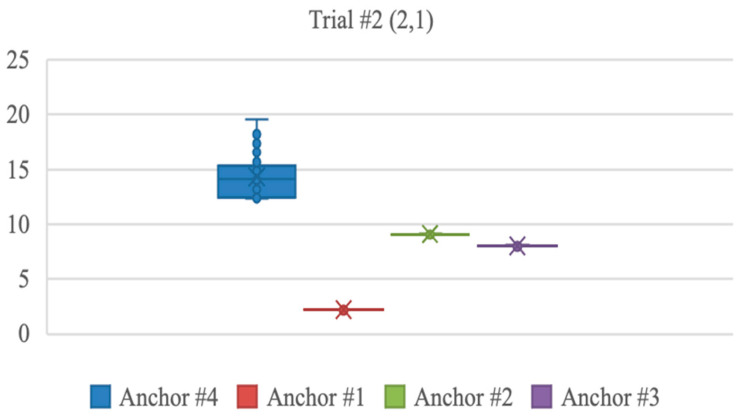
Distance measurement distribution for each anchor.

The distribution of distance measurements for trial 2 (2,1) is illustrated in Figure 7. It was observed that the anchor located at (10,10) provided a wide range of distance detections. Although the actual distance between this anchor and the stationary tag was 12.04 m, the distance measurement reached 19.54 m. This error in measurement could be attributed to the proximity between the tag and the anchor. It was observed that the error increased when the distance between the anchor and tag was more than 10 m.

**Figure 8:**
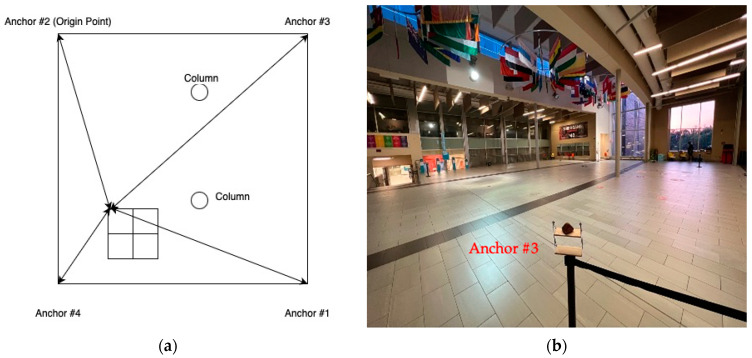
(**a**) A tag placed at one of the reference points; (**b**) A UWB anchor position.

**Figure 9:**
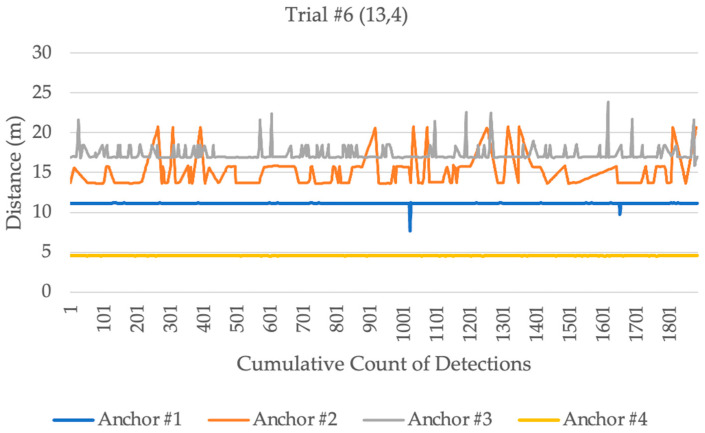
Distance measurements for 1 min from each anchor.

The study area was extended to 15 m by 15 m on 7 July 2023 to investigate the results of the stationary experiment for 1 min interval on each coordinate, as shown in Figure 8. The tag was at a 1 m distance from the floor, while the anchors were at 1.10 m. Table 3 illustrates the mean average error in distance. Anchor #2 had five failures out of nine trials; this can be due to the orientation of the anchor [22]. At trials 6 and 8, anchor# 3 showed a high average error at the distances 0.38 m and 0.29 m, respectively, because of potential multipath delay [23]. Figure 9 demonstrates the distance ranging from each anchor for a 1 min interval. These results could be influenced by many factors, including multipath signal delay [24,25]. Finally, trial 7 experienced failures where the tag did not record any detection. This could be as a result of restarting the tag.

### 3.2. Ranging Experiment in Dual Communication Mode

There are many reasons that can potentially cause ranging errors. Tiemann et al. [26] discussed the source of distance-ranging errors, such as clock drift, frequency drift, LOS/NLOS, multi-user interference, or malicious attacks. The UWB dual mode was tested, where initiator and responder switches were among the initiator and responder roles. The initiator and responder changed their role every 500 milliseconds with 20% randomization to avoid signal interference.

**Figure 10:**
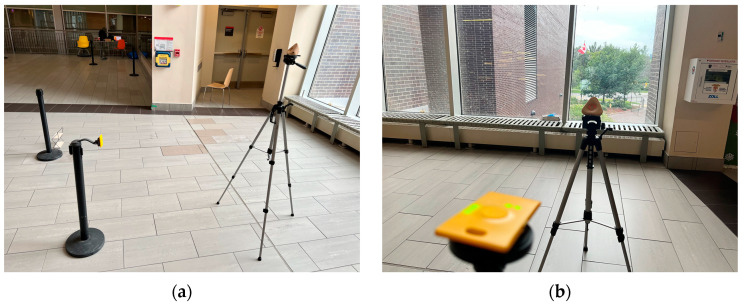
(**a**) A tag placed at 1 m height vertically; (**b**) A tag placed at 1 m height horizontally.

The first experiment was placing the anchor at 1.65 m height and the tag at 1 m. The experiment investigated two orientation scenarios, such as placing the tag horizontally and vertically, as shown in Figure 10. Then, the study looked into placing the anchor and the tag at the same height (1 m), as illustrated in Figure 10b.

**Figure 11:**
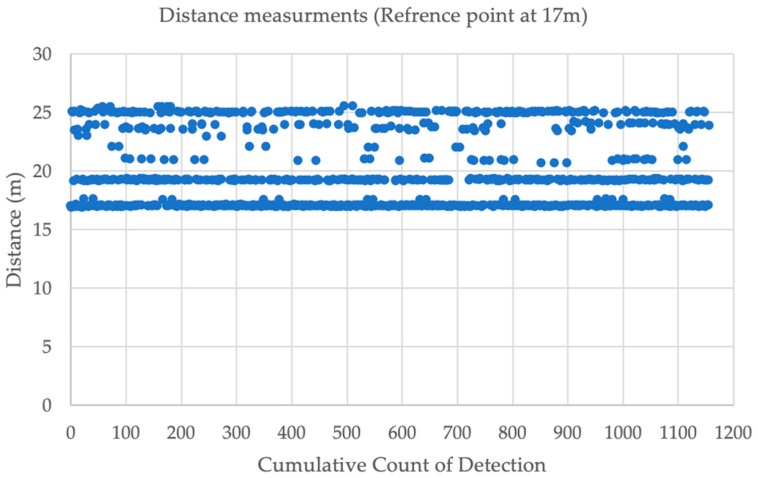
Distance measurements at point 17 m.

Table 4 and Table 5 show the distance measurement when placing the tag at various distance ranges to explore accuracy at each point for a 1 min interval. The anchor was at 1.65 m height, and the tag was at 1 m height. The UWB distance ranging measurements were compared to the oblique distance. The experiment found at 17 m that the MAPE in the distance was 2.71 m. Figure 10 shows the distance measurements at point 17 m. This issue occurred due to multipath because of the consistent distance fluctuation as shown in Figure 11.

**Figure 12:**
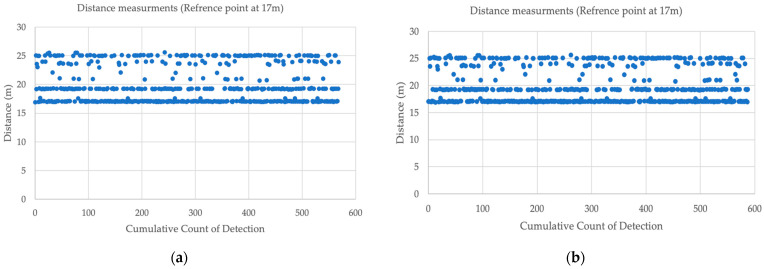
(**a**) Distance measurements when the tag was the initiator; (**b**) Distance measurements when the anchor was the initiator.

This study looked in depth to identify the issue. For instance, because of the dual mode (changing the initiator and the responder in their role), one device could produce this distance of measurements. However, Figure 12 shows that both devices had a multipath delay.

The number of detections in most cases was found when the tag moved away from the anchor. The study found that the distance ranging of the tag when placed horizontally topped after 10 m. The number of detections increased by about one-third at 1 m and 3 m. Table 6 and Table 7 illustrate the distance ranging measurements when the tag and anchor were at 1 m height. There was a correlation between the distance ranging and the number of detections, where the number of detections decreased over distance ranging. During the distance ranging from 20 m, the MAPE in distance reached 3 m. There were no distance measurements when the tag was placed horizontally. Chansamood et al. [27] investigated the impact of antenna orientation between the initiator and responder. The authors claimed that the highest range could be achieved when the initiator and receiver were placed vertically. Moreover, the worst cases were when the initiator and receiver were both placed horizontally over one of them. As a result, the orientation of the antenna can influence the error in distance ranging.

### 3.3. Dynamic Positioning Experiment

**Figure 13:**
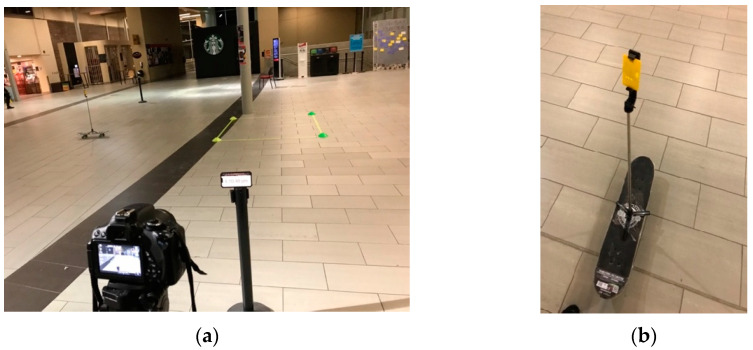
(**a**) Camera for recording the experiments, (**b**) UWB tag on a skateboard.

This study aimed to evaluate the accuracy of UWB technology through dynamic experiments using anchors and tags. Numerous experiments were conducted to measure the position and movement of moving objects. In the first set of experiments, four anchors were fixed in a tripod location in a study area measuring 10 m by 10 m. Two tags were attached to selfie sticks, with one held by a pedestrian and the other attached to a skateboard. The tag was placed at the center of the skateboard, as shown in Figure 13, and the other tag was held by the pedestrian at a height of 2 m to avoid any line of sight issues. A conflict zone measuring 2 m by 2 m was outlined to track the positions of the tags. Upon enabling UWB on the anchors and tags, the tags began receiving and storing distance measurements and timestamps.

The primary objective of this experiment was to investigate the efficacy of UWB technology when estimating PET. This traffic conflict indicator has been widely used to gauge the proximity of pedestrians and vehicles to identify traffic-related conflicts [15]. To estimate PET, the study focused on tracking a single UWB tag. The experiments were conducted on 31 January 2023, 7 February 2023, and 15 February 2023. During the first experiment, the study area measured 10 m by 10 m, with a conflict area of 1 m by 1 m located between (3,2) and (4,3). However, upon analyzing the UWB tag data, it was observed that the tag did not enter the zone due to its small size. Therefore, in the experiment conducted on 7 February 2023, the conflict zone was expanded to 2 m by 2 m to evaluate the positioning accuracy of UWB tags.

More investigations were conducted on 18 and 23 June 2023 to apply dual model double-sided two-way communication. However, time synchronization was a challenge because the clock of each device was different from the other. This issue was also discussed in Pérez-Solano et al.’s research [28]. Therefore, on 7 July 2023, one tag four anchor experiments were conducted to investigate time occupancy without signal interference, collision in transmission, and the time synchronization issue. The experiments on 2 and 7 July 2023 explored a study area of 8 m by 8 m and 15 m by 15 m. The results of dynamic experiments are discussed in the following section.

## 4. Results

**Figure 14:**
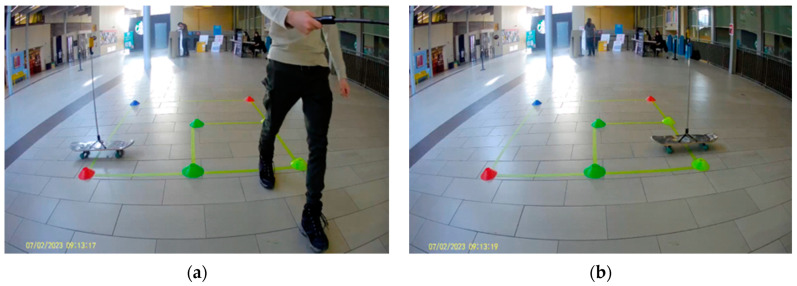
(**a**) The tag enters the conflict area, (**b**) The tag exits the conflict area.

The movement of the UWB tag entering and exiting the conflict area is shown in Figure 14. The study included 50 trials conducted on 7 February 2023, out of which 32 trials were analyzed and compared to ground truth data using a video camera. The study area was 10 m by 10 m, and the conflict area was 2 m by 2 m, as indicated in Figure 12. The conflict zone was located between coordinates (1,3) to (3,5). Table 8 displays a comparison between the timestamps of ground truth data and tags when entering and exiting the conflict zone for one tag (skateboard). Trials 6 and 8 revealed that the pedestrian and skateboard were inside the conflict zone, indicating a safety hazard when estimating post-encroachment time. In addition, some trials resulted in “No Detection” due to issues in LTE communication, and the anchors were only measuring detections for one tag. The mean absolute difference was 0.43 s between the ground truth data and UWB technology. Consequently, additional experiments were conducted on 15 February 2023 by increasing the number of anchors to avoid miscommunication between the anchors and the tag.

**Figure 15:**
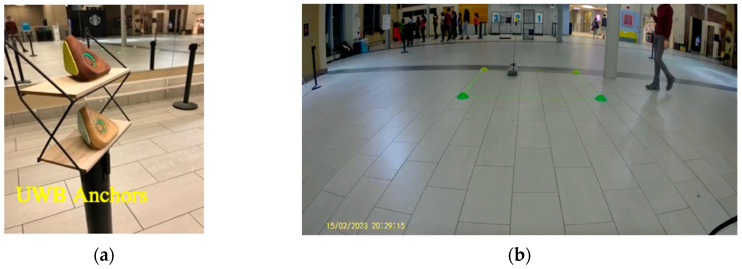
(**a**) The UWB anchors, (**b**) The skateboard UWB tag and pedestrian UWB tag.

On 15 February 2023, this study aimed to improve UWB technology’s accuracy by increasing the number of anchors, as depicted in Figure 15. A conflict zone of 2 m by 2 m was placed in the center of the study area, between (4,4) and (6,6), with the skateboard moving approximately at the centerline of the conflict zone. The experiment started with the pedestrian and skateboard at (10,5) and (5,10) respectively. The pedestrian moved from point (10,5) to (0,5), while the skateboard was pulled by a rope from point (5,10) to (5,0). The ground truth data were obtained by estimating the time in and out using a video camera, while the UWB tag’s time in and out was stored in the cloud. One anchor failed to be detected during the experiment. In some trials, the tag did not upload measurements to the cloud due to miscommunication, resulting in failed trials identified as “No Detection”. The mean absolute difference was 0.68 s. The average means absolute difference from the February 7 and February 15 experiments combined was 0.55 s.

Additional experiments were conducted to analyze the single tag with four anchors on 2 and 7 July 2023 without challenges related to time synchronization on dual mode or collision in transmission due to multiple devices attempting to transmit signals simultaneously, as mentioned earlier. The 2 July experiment analyzed time occupancy from ground truth data obtained from a camera and UWB estimation. The study area was 8 m by 8 m, and a conflict area was outlined on the floor at (3,3) to (5,5). The first 11 trials with four anchors estimated distance measurements. However, one anchor failed to continue recording distance measurements, and the results are shown in Table 9. Therefore, the first 11 trials applied multilateration and then applied trilateration for the rest of the trials. This study found that the average absolute difference in time occupancy was 0.05s. Because of the accuracy in the average absolute difference in time occupancy, the 7 July experiment was conducted. The experiment looked at the time occupancy with a study area of 8 m by 8 m. Table 10 shows the results from a study area of 8 m by 8 m for 7 July. The conflict zone was located at (3,3) to (5,5). Four anchors were multiliterate to produce the positioning of the tag. Table 11 shows raw data from trial 4 from Table 10 when the tag entered and exited the conflict zone. Moreover, the raw data show the distance measurements from each anchor with every time increment.

**Figure 16:**
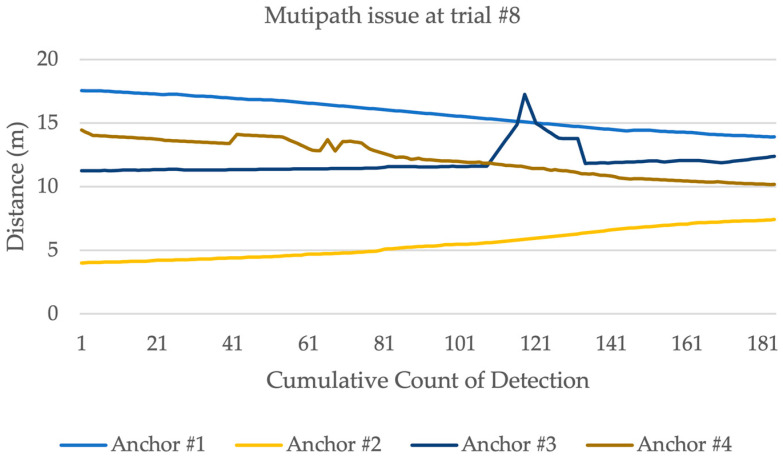
Distance measurements from each anchor in trial #8.

After exploring the time occupancy in the study area of 8 m by 8 m, the research investigated the time occupancy in the 15 m by 15 m study area with the same conflict zone (3,3) to (5,5). Table 12 demonstrates the results in time occupancy. The results show one failure in detection. Moreover, one of the anchors at trial 12 stopped recording distance measurements; therefore, Trilateration was applied for that trial. There are some trials that showed a high absolute error in time occupancy, such as trials 4, 8, 13, and 17 in Table 12. After looking into the data, the study found that speed filtering (when removing data points related to speed relative > 5 m/s) was not applied to Table 12, which caused inaccurate localization. Therefore, Table 13 demonstrates the results after applying the filter. Trial 8 on Table 12 shows the maximum absolute error difference in time occupancy of 0.76. Figure 16 shows the distance measurements from each anchor. Anchor #3 was affected by the multipath issue, which resulted in an error in localization.

## 5. Discussion

Although UWB technology offers many advantages, there are many challenges and considerations that must be considered when implementing and designing a UWB positioning system. First, the orientation of UWB anchor antennas significantly impacts tracking accuracy. The anchor antenna’s orientation should be toward the center of the tracking area in order to overlap with the radiation pattern [22]. Many studies have looked into optimizing polarization and radiation. Polarization refers to the orientation of the electric field in an electromagnetic wave. For instance, a study by Alfakhri and Zhang et al. [29,30] investigated a Multiple-Input-Multiple-Output (MIMO) antenna design to accomplish dual-polarization operation and a Defected Ground Structure (DGS). Moreover, the design showed a stable radiation pattern. Therefore, by exploring antennas that support multiple or dual polarizations, UWB technology can receive signals from various orientations.

Many studies have explored the placement of antennas to optimize radiation and polarization. For instance, Fortes et al. [31] found that the appropriate placement of antennas depended on the height of the antennas. This study investigated the antennas from a height of 1.3 m to 2.5 m, which resulted in a declining mean tracking error from 47 cm to 26 cm. Therefore, more investigations are needed to investigate the optimum height for an antenna in an outdoor environment.

The accuracy of UWB technology can be impacted by the NLOS effect. Research was conducted by Ansaripour et al. [11] to overcome this issue by increasing the number of tags surrounding the object. Another research applied machine learning algorithms to reduce the NLOS effects [32]. For example, this study used many ML techniques, such as K-Nearest Neighbors (k-NN), Support Vector Machine (SVM), Decision Tree (DT), Naïve Bayes (NB), and Neural Network (NN) to classify and reduce the localization error. A confusion matrix was used to investigate the performance of these techniques. This study found that, in the case of LOS, the True Positives (TP) = 983 and False Negative (FN) = 17, the precision, recall, and accuracy were 98.9%, 98.3%, and 97.5%, respectively. Finally, in the case of NLOF, the TP = 890, and FN = 110 with the average running time of NN was 0.0606.

The failed detections can be minimized by storing the data in the tag’s storage. Moreover, the size of detections can consume battery life. Thus, the tags can be connected to a power source to maintain the detection rate. One of the benefits of UWB technology is to provide high-accuracy positioning. Therefore, the pedestrian tag should be placed higher to avoid the NLOS and measure accurate positioning.

Two critical challenges were encountered during the experiments: multipath interference and NLOS. These challenges could potentially impact accuracy in the field of traffic conflict techniques (TCT). For instance, both challenges can result in inaccurate positioning, which leads to an inaccurate conflict prediction. This study was conducted to evaluate the accuracy of UWB when calculating time occupancy in an indoor environment. Therefore, this could lead to a better understanding of the limitations of this technology and the preparation for successful deployment in an outdoor environment. It is hoped that the findings gained from the indoor trails will help mitigate challenges in an outdoor setting and ensure more safer and reliable technology.

Range and interference limitations can impact positioning accuracy. Hence, it is crucial to optimize the system parameters, including the study size, the height of the anchors, and signal filtering. More studies are needed on UWB technology outdoors, such as various weather conditions and using an actual vehicle with a skateboard to design an experiment that reflects actual road users. The communication between the anchors and tags should be optimized for consistent, accurate positioning. Finally, time measurement must be synchronized with the actual time to have real-time positioning and estimate post-encroachment time.

## 6. Conclusions

This research evaluated UWB technology in potentially estimating post-encroachment time. As UWB technology continues to improve and become more widely available, it is expected to play a significant role in ensuring efficient and safe transportation systems. This study showed a high accuracy in time occupancy, which reached an average of 0.06 s in the absolute mean error for single-tag experiments in an 8 m by 8 m study area. This study found that the accuracy of time occupancy at 15 m by 15 m increased the time occupancy error to 0.22 s. This research looked at static and dynamic positioning experiments. The research found that the range accuracy could be affected by the range and signal interference from the second tag. Many challenges were discussed in this study that needed to be investigated and an accurate and reliable UWB system was achieved. As UWB technology is improving, many traffic conflict applications can be utilized and enhance transportation systems to accomplish sustainability. Future work should estimate post-encroachment time using dual mode and be able to communicate with multiple tags. Also, time synchronization should be considered with the dual mode by broadcasting the time from one anchor to other UWB devices, including tags and anchors.

## Figures and Tables

**Table 1:** The errors in coordinates at each reference point.

Trial	Coordinates	Error *x*	Error *y*
1	(1,3)	0.25	0.01
2	(2,3)	0.03	0.03
3	(3,3)	0.02	0.09
4	(1,4)	0.22	0.05
5	(2,4)	0.01	0.04
6	(1,5)	0.04	0.01

**Table 2:** Sample of raw data.

ID#	Timestamp	Trial#	(x, y)
c1	1 676 505 518 024	2	(1.90, 0.99)
c1	1 676 505 518 035	2	(1.90, 0.99)
c1	1 676 505 518 211	2	(1.88, 0.93)
c1	1 676 505 518 235	2	(1.88, 0.93)
c1	1 676 505 518 266	2	(1.89, 0.94)
c1	1 676 505 518 291	2	(1.89, 0.95)

**Table 3:** The mean average error (m) in distance for each anchor.

Trial	Coordinates	Anchor#1	Anchor#2	Anchor#3	Anchor#4
1	(11,3)	0.05	5.68	0.03	0.09
2	(12,3)	0.07	-	0.03	0.03
3	(13,3)	0.12	-	0.06	0.11
4	(11,4)	0.10	-	0.06	0.10
5	(12,4)	0.07	1.35	0.04	0.07
6	(13,4)	0.05	1.62	0.38	0.09
7	(11,5)	-	-	-	-
8	(12,5)	0.01	0.07	0.29	0.10
9	(13,5)	0.02	-	0.06	0.11
Average error		0.06	2.18	0.11	0.08

**Table 4:** Distance ranging when the tag is vertical.

Reference Point (m)	MAPE (M)	Number of Detections
1	0.06	1537
3	0.02	1686
5	0.01	1423
10	0.02	1273
15	0.02	309
16	0.03	200
17	2.71	1123
17 (Repeated)	2.71	156
18	0.05	490
19	0.05	1699
20	0.04	1770

**Table 5:** Distance ranging when the tag is horizontal.

Reference Point (m)	MAPE (M)	Number of Detections
1	0.03	2159
3	0.04	2080
5	0.06	1385
10	0.04	480
15	-	-
16	-	-
17	-	-
18	-	-
19	-	-
20	-	-

**Table 6:** Distance ranging when the tag is vertical.

Reference Point (m)	MAPE (M)	Number of Detections
1	0.07	1968
3	0.05	1678
5	0.03	1523
10	0.02	1172
15	0.07	1815
16	0.07	499
17	0.09	779
18	0.09	673
19	0.11	365
20	3.00	104

**Table 7:** Distance ranging when the tag is horizontal.

Reference Point (m)	MAPE (M)	Number of Detections
1	0.18	1757
3	0.04	1259
5	0.14	1294
10	1.89	992
15	-	-
16	-	-
17	-	-
18	-	-
19	-	-
20	-	-

**Table 8:** The absolute differences between UWB-based and ground truth data.

	Ground Truth		UWB Tag		Absolute Difference (s)
Trial#	Time In	Time Out	Time In	Time Out	
1	09:06:19.333	09:06:20.733	09:06:22.394	09:06:23.353	0.44
2	09:07:37.500	09:07:41.833	09:07:40.716	09:07:44.107	0.94
3	09:09:42.866	09:09:43.833	09:09:45.732	09:09:46.740	0.04
4	09:10:42.566	09:10:44.240	09:10:45.221	09:10:46.865	0.03
5	09:11:25.866	09:11:27.700	09:11:29.228	09:11:30.420	0.64
6 *	09:12:19.433	09:12:21.300	09:12:21.270	09:12:24.633	1.50
7	09:13:17.000	09:13:19.233	09:13:21.008	09:13:22.206	1.03
8 *	09:14:37.066	09:14:38.066	09:14:39.027	09:14:40.989	0.96
9	09:15:31.800	09:15:33.000	No Detection	No Detection	No Detection
10	09:16:47.200	09:16:48.300	09:16:49.959	09:16:51.084	0.02
11	09:17:50.433	09:17:51.833	09:17:53.182	09:17:55.055	0.47
12	09:19:01.200	09:19:02.600	09:19:03.484	09:19:05.366	0.48
13	09:20:33.066	09:20:34.100	09:20:35.461	09:20:36.828	0.33
14	09:21:30.866	09:21:32.400	09:21:33.276	09:21:34.753	0.06
15	09:24:00.833	09:24:01.900	09:24:04.143	09:24:04.668	0.54
16	09:24:59.666	09:25:01.200	No Detection	No Detection	No Detection
17	09:26:17.600	09:26:18.866	09:26:19.843	09:26:21.432	0.32
18	09:27:23.100	09:27:24.233	No Detection	No Detection	No Detection
19	09:28:13.133	09:28:14.100	09:28:15.923	09:28:16.572	0.32
20	09:29:05.166	09:29:06.233	No Detection	No Detection	No Detection
21	09:31:24.666	09:31:25.933	No Detection	No Detection	No Detection
22	09:33:02.133	09:33:03.566	No Detection	No Detection	No Detection
23	09:36:44.133	09:36:45.533	09:36:47.350	09:36:48.260	0.49
24	09:37:34.733	09:37:35.766	09:37:37.348	09:37:38.467	0.09
25	09:38:27.266	09:38:28.833	09:38:30.279	09:38:31.500	0.35
26	09:39:23.800	09:39:27.000	No Detection	No Detection	No Detection
27	09:40:43.100	09:40:45.000	09:40:46.036	09:40:47.759	0.18
28	09:42:01.633	09:42:03.333	09:42:04.166	09:42:05.730	0.14
29	09:42:59.200	09:43:00.100	09:43:02.610	09:43:03.699	0.19
30	09:44:16.066	09:44:17.333	No Detection	No Detection	No Detection
The mean absolute difference					0.43 s

* The trials 6 and 8 revealed that the pedestrian and skateboard were inside the conflict zone.

**Table 9:** The time occupancy from ground truth data and UWB estimation for the 8 m by 8 m study area (2 July 2023).

Trial#	UWB Time In	UWB Time Out	GT Time Occupancy	UWB Time Occupancy	Absolute Difference in Time Occupancy
1	02:10:27.755	02:10:28.400	0.67	0.65	0.02
2	02:12:08.528	02:12:09.466	0.80	0.94	0.14
3	02:13:06.176	02:13:06.799	0.63	0.62	0.01
4	02:14:06.491	02:14:07.314	0.87	0.82	0.04
5	02:15:05.809	02:15:06.636	0.83	0.83	0.01
6	02:16:08.662	02:16:09.431	0.73	0.77	0.04
7	02:17:06.172	02:17:06.853	0.67	0.68	0.01
8	02:18:03.608	02:18:04.323	0.70	0.72	0.02
9	02:19:04.693	02:19:05.501	0.77	0.81	0.04
10	02:20:04.594	02:20:05.488	0.90	0.89	0.01
11	02:21:04.269	02:21:04.907	0.60	0.64	0.04
12	02:22:04.958	02:22:05.587	0.67	0.63	0.04
13	02:23:04.383	02:23:05.133	0.53	0.75	0.22
14	02:24:05.809	02:24:06.591	0.80	0.78	0.02
15	02:25:05.229	02:25:05.987	0.77	0.76	0.01
16	02:26:08.869	02:26:09.628	0.70	0.76	0.06
17	02:27:04.890	02:27:05.504	0.70	0.61	0.09
18	02:28:06.825	02:28:07.866	0.97	1.04	0.07
19	02:29:07.726	02:29:08.446	0.70	0.72	0.02
20	02:30:05.430	02:30:06.173	0.70	0.74	0.04
21	02:32:05.622	02:32:06.657	1.00	1.03	0.03
22	02:33:18.356	02:33:19.177	0.83	0.82	0.01
23	02:34:07.544	02:34:09.678	2.03	2.13	0.10
24	02:35:04.900	02:35:05.638	0.73	0.74	0.00
25	02:36:09.645	02:36:10.687	1.07	1.04	0.02
26	02:37:04.204	02:37:04.821	0.67	0.62	0.05
27	02:38:05.722	02:38:06.506	0.77	0.78	0.02
28	02:39:20.274	02:39:20.796	0.63	0.52	0.11
29	02:41:11.115	02:41:11.940	0.77	0.83	0.06
30	02:42:12.129	02:42:12.773	0.67	0.64	0.02
31	02:43:12.010	02:43:12.643	0.67	0.63	0.03
32	02:44:05.504	02:44:06.193	0.73	0.69	0.04
Average					0.05 s

**Table 10:** The time occupancy from ground truth data and UWB estimation for the 8 m by 8 m study area (7 July 2023).

Trial#	UWB Time In	UWB Time Out	GT Time Occupancy	UWB Time Occupancy	Absolute Difference in Time Occupancy
1	08:04:20.954	08:04:21.877	0.97	0.92	0.04
2	08:06:03.193	08:06:03.782	0.57	0.59	0.02
3	08:07:04.950	08:07:05.542	0.70	0.59	0.10
4	08:08:24.503	08:08:25.250	0.90	0.75	0.15
5	08:09:11.094	08:09:11.915	0.90	0.82	0.07
6	08:10:03.628	08:10:04.441	0.90	0.81	0.08
7	08:11:02.290	failure	1.03	-	-
8	failure	failure	0.87	-	-
9	08:13:06.773	08:13:07.947	1.27	1.17	0.09
10	failure	failure	0.83	-	-
11	08:15:03.740	08:15:04.723	1.03	0.98	0.05
12	08:18:16.267	08:18:17.224	1.00	0.96	0.04
13	08:20:03.947	08:20:04.880	1.03	0.93	0.10
14	08:21:10.613	08:21:11.375	0.63	0.76	0.12
15	08:22:05.088	08:22:05.711	0.67	0.62	0.04
16	08:23:14.469	08:23:15.193	0.80	0.72	0.07
17	failure	failure	1.1	-	-
18	failure	failure	1.7	-	-
19	08:32:03.078	08:32:03.924	0.8	0.85	0.04
20	failure	failure	1.33	-	-
21	08:34:06.721	00:34:07.700	1.13	1.01	0.12
Average					0.08 s

**Table 11:** Raw data from trial #4 (7 July 2023).

Timestamp	Anchor#1	Anchor#2	Anchor#3	Anchor#4	[*x*, *y*]
20:08:24.448	4.79	6.58	6.31	5.04	[5.08, 4.17]
20:08:24.458	4.80	6.56	6.26	5.03	[5.05, 4.18]
20:08:24.468	4.80	6.55	6.21	5.02	[5.03, 4.18]
20:08:24.472	4.81	6.53	6.22	5.01	[5.03, 4.17]
20:08:24.481	4.82	6.52	6.24	5.03	[5.02, 4.16]
20:08:24.489	4.84	6.51	6.25	5.05	[5.01, 4.16]
20:08:24.503	4.85	6.48	6.27	5.07	[4.99, 4.14]
20:08:24.515	4.87	6.45	6.21	5.09	[4.95, 4.15]
20:08:24.523	4.92	6.43	6.16	5.11	[4.90, 4.15]
20:08:24.533	4.96	6.39	6.11	5.13	[4.84, 4.15]
20:08:24.646	5.00	6.35	6.05	5.15	[4.79, 4.15]
20:08:24.678	5.04	6.32	5.99	5.28	[4.70, 4.19]
20:08:24.683	5.09	6.28	5.93	5.41	[4.60, 4.23]
20:08:24.693	5.10	6.25	5.87	5.53	[4.52, 4.28]
20:08:24.697	5.12	6.21	5.81	5.57	[4.47, 4.30]
20:08:24.723	5.13	6.18	5.79	5.60	[4.43, 4.30]
20:08:24.729	5.15	6.14	5.77	5.65	[4.39, 4.30]
20:08:24.745	5.16	6.10	5.74	5.69	[4.35, 4.31]
20:08:24.750	5.18	6.07	5.71	5.73	[4.30, 4.32]
20:08:24.756	5.20	6.05	5.67	5.77	[4.27, 4.33]
20:08:24.760	5.21	6.03	5.64	5.79	[4.23, 4.34]
20:08:24.780	5.23	6.01	5.63	5.82	[4.21, 4.34]
20:08:24.784	5.25	5.99	5.61	5.84	[4.18, 4.34]
20:08:24.789	5.27	5.98	5.60	5.86	[4.16, 4.34]
20:08:24.793	5.29	5.97	5.58	5.89	[4.13, 4.35]
20:08:24.811	5.30	5.96	5.55	5.91	[4.10, 4.36]
20:08:24.816	5.33	5.95	5.52	5.94	[4.07, 4.37]
20:08:24.825	5.35	5.93	5.49	5.96	[4.04, 4.37]
20:08:24.846	5.37	5.91	5.46	5.98	[4.01, 4.37]
20:08:24.856	5.38	5.89	5.43	6.01	[3.97, 4.38]
20:08:24.859	5.40	5.87	5.41	6.03	[3.94, 4.38]
20:08:24.877	5.42	5.85	5.39	6.05	[3.91, 4.38]
20:08:24.884	5.44	5.83	5.36	6.07	[3.88, 4.38]
20:08:24.894	5.47	5.82	5.34	6.08	[3.86, 4.38]
20:08:24.909	5.49	5.82	5.32	6.10	[3.84, 4.39]
20:08:24.919	5.50	5.82	5.30	6.12	[3.82, 4.40]
20:08:24.925	5.51	5.80	5.27	6.14	[3.79, 4.40]
20:08:24.940	5.52	5.77	5.25	6.15	[3.77, 4.40]
20:08:24.950	5.53	5.75	5.23	6.16	[3.75, 4.40]
20:08:24.954	5.53	5.75	5.21	6.16	[3.73, 4.41]
20:08:24.960	5.54	5.74	5.20	6.17	[3.73, 4.41]
20:08:24.974	5.55	5.74	5.20	6.19	[3.72, 4.41]
20:08:24.980	5.56	5.73	5.20	6.20	[3.70, 4.41]
20:08:24.985	5.57	5.72	5.19	6.21	[3.69, 4.41]
20:08:24.995	5.58	5.70	5.16	6.22	[3.66, 4.41]
20:08:25.008	5.60	5.69	5.13	6.24	[3.64, 4.42]
20:08:25.015	5.61	5.68	5.10	6.25	[3.61, 4.42]
20:08:25.027	5.63	5.66	5.06	6.26	[3.59, 4.43]
20:08:25.038	5.64	5.64	5.03	6.28	[3.56, 4.43]
20:08:25.047	5.65	5.63	5.00	6.30	[3.53, 4.44]
20:08:25.050	5.67	5.63	4.97	6.32	[3.50, 4.46]
20:08:25.061	5.68	5.62	4.95	6.35	[3.49, 4.46]
20:08:25.070	5.69	5.62	4.94	6.34	[3.48, 4.46]
20:08:25.077	5.70	5.62	4.93	6.34	[3.47, 4.46]
20:08:25.081	5.71	5.61	4.92	6.34	[3.46, 4.46]
20:08:25.092	5.72	5.60	4.89	6.34	[3.45, 4.46]
20:08:25.108	5.74	5.60	4.87	6.37	[3.42, 4.47]
20:08:25.109	5.77	5.59	4.85	6.40	[3.39, 4.48]
20:08:25.124	5.80	5.58	4.83	6.43	[3.35, 4.48]
20:08:25.159	5.83	5.57	4.81	6.55	[3.29, 4.52]
20:08:25.168	5.86	5.56	4.79	6.60	[3.25, 4.53]
20:08:25.179	5.88	5.55	4.76	6.65	[3.21, 4.54]
20:08:25.192	5.91	5.54	4.74	6.70	[3.17, 4.55]
20:08:25.203	5.94	5.53	4.72	6.72	[3.14, 4.55]
20:08:25.214	5.97	5.51	4.70	6.75	[3.10, 4.55]
20:08:25.228	6.01	5.50	4.66	6.78	[3.06, 4.56]
20:08:25.238	6.04	5.49	4.62	6.81	[3.02, 4.57]
20:08:25.250	6.08	5.46	4.59	6.85	[2.97, 4.57]
20:08:25.401	6.11	5.44	4.50	6.89	[2.91, 4.60]
20:08:25.411	6.14	5.42	4.40	7.02	[2.81, 4.65]

**Table 12:** The time occupancy from ground truth data and UWB estimation for 15m-by-15m study area (7 July 2023).

Trial#	UWB Time In	UWB Time Out	GT Time Occupancy	UWB Time Occupancy	Absolute Difference in Time Occupancy
1	09:24:23.553	09:24:24.000	0.63	0.45	0.18
2	09:26:00.581	09:26:01.338	1.27	0.76	0.51
3	failure	-	1.17	-	-
4	09:28:34.635	09:28:34.847	1.20	0.21	0.98
5	09:29:23.000	09:29:24.000	1.17	1.00	0.16
6	09:30:38.567	09:30:39.089	0.97	0.52	0.44
7	09:31:52.665	09:31:53.025	0.83	0.36	0.47
8	09:32:49.400	09:32:50.065	1.43	0.67	0.76
9	09:33:34.748	09:33:35.720	1.20	0.97	0.22
10	09:34:28.536	09:34:29.540	1.10	1.00	0.09
11	09:35:20.547	09:35:21.171	1.20	0.62	0.57
12	09:36:13.721	09:36:14.882	1.30	1.16	0.13
13	09:37:13.378	09:37:13.950	1.17	0.57	0.59
14	09:38:15.596	09:38:16.798	1.27	1.20	0.06
15	09:39:08.722	09:39:09.512	1.17	0.79	0.37
16	09:40:23.705	09:40:24.733	1.13	1.03	0.10
17	09:41:22.153	09:41:22.689	1.13	0.54	0.59
18	09:42:17.233	09:42:18.164	1.1	0.93	0.16
19	09:43:17.945	09:43:18.886	1.03	0.94	0.09
Average					0.36 s

**Table 13:** The time occupancy from ground truth data and UWB estimation for 15 m-by-15 m study area (7 July 2023—after applying speed filter).

Trial#	UWB Time In	UWB Time Out	GT Time Occupancy	UWB Time Occupancy	Absolute Difference in Time Occupancy
1	09:24:23.553	09:24:24.000	0.63	0.45	0.18
2	09:26:00.581	09:26:01.338	1.27	0.76	0.51
3	failure	-	1.17	-	-
4	09:28:34.635	09:28:35.294	1.20	0.66	0.54
5	09:29:23.000	09:29:24.162	1.17	1.00	0.01
6	09:30:38.567	09:30:39.089-	0.97	0.52	0.44
7	09:31:52.665	09:31:53.025-	0.83	0.36	0.47
8	09:32:49.400	09:32:50.065-	1.43	0.67	0.76
9	09:33:34.748	09:33:35.720-	1.20	0.97	0.22
10	09:34:28.536	09:34:29.540-	1.10	1.00	0.09
11	09:35:20.547	09:35:21.692	1.20	1.14	0.055
12	09:36:13.721	09:36:14.882-	1.30	1.16	0.13
13	09:37:13.378	09:37:14.451	1.17	1.07	0.09
14	09:38:15.596	09:38:16.798-	1.27	1.20	0.06
15	09:39:08.722	09:39:09.895	1.17	1.17	0.00
16	09:40:23.705	09:40:24.733-	1.13	1.03	0.10
17	09:41:22.153	09:41:23.185	1.13	1.03	0.10
18	09:42:17.233	09:42:18.164	1.1	0.93	0.16
19	09:43:17.945	09:43:18.886	1.03	0.94	0.09
Average					0.22 s

## Data Availability

The data presented in this study pertaining to the wireless sensor measurements are available on request from the corresponding author. The data are not publicly available due to equipment privacy and security reasons. Other data may not be shared due to privacy reasons.
